# PAIN MANAGEMENT FOR OLDER ADULTS LIVING WITH DEMENTIA IN NURSING HOME COMMUNITIES

**DOI:** 10.1093/geroni/igae098.1805

**Published:** 2024-12-31

**Authors:** Barbara Resnick

**Affiliations:** Chair

## Abstract

Pain is a commonly reported symptom among older adults living with dementia with 30 to 80% of those in nursing homes experiencing pain. For over 50% of these individuals the pain is moderate to severe. Pain often results in a decline in function and quality of life, exacerbates behavioral symptoms associated with dementia, contributes to delirium and increases the risk of emergency room visits. For those with moderate to severe dementia verbal reporting of pain may not be reliable. Multiple systematic reviews concluded that self-report tools should be used when possible and observational measures used for supplementation of self-report. Other challenges associated with pain assessment, diagnosis and management among residents living with dementia include: (1) variations in pain with pain being more intense some days and during certain activities; (2) acceptance of pain as normal; (3) fear of pharmacological interventions; (4) lack of belief in nonpharmacological approaches; (5) lack of knowledge; (6) communication challenges between providers; and (7) desensitization to pain as experienced by residents and observed by staff. The State Operations Manual for Long Term Care, specifically F-Tag F697, requires that nursing homes must ensure that pain management is provided to residents who require such services. This symposium provides an overview of the Pain Management Clinical Practice Guideline (CPG) for nursing homes residents, results from pilot testing of a pragmatic implementation approach to help integrate use of the CPG into the community, and a review of the facility factors associated with pain among nursing home residents living with dementia.

